# Candidalysin Is a Potent Trigger of Alarmin and Antimicrobial Peptide Release in Epithelial Cells

**DOI:** 10.3390/cells9030699

**Published:** 2020-03-12

**Authors:** Jemima Ho, Don N. Wickramasinghe, Spyridoula-Angeliki Nikou, Bernhard Hube, Jonathan P. Richardson, Julian R. Naglik

**Affiliations:** 1 Centre for Host-Microbiome Interactions, Faculty of Dentistry, Oral & Craniofacial Sciences, King’s College London, London SE1 1UL, UK; don.wickramasinghe@kcl.ac.uk (D.N.W.); spyridoula.nikou@crick.ac.uk (S.-A.N.); jonathan.richardson@kcl.ac.uk (J.P.R.); julian.naglik@kcl.ac.uk (J.R.N.); 2 Protein Phosphorylation Lab, The Francis Crick Institute, London NW1 1AT, UK; 3 Department of Microbial Pathogenicity Mechanisms, Leibniz Institute for Natural Product Research and Infection Biology–Hans Knöll Institute (HKI), 07745 Jena, Germany; bernhard.hube@hki-jena.de; 4 Institute of Microbiology, Friedrich Schiller University, 07745 Jena, Germany

**Keywords:** *Candida*, candidalysin, antimicrobial peptide, alarmin, ATP, defensin

## Abstract

Host released alarmins and antimicrobial peptides (AMPs) are highly effective as antifungal agents and inducers. Whilst some are expressed constitutively at mucosal tissues, the primary site of many infections, others are elicited in response to pathogens. In the context of *Candida albicans*, the fungal factors inducing the release of these innate immune molecules are poorly defined. Herein, we identify candidalysin as a potent trigger of several key alarmins and AMPs known to possess potent anti-*Candida* functions. We also find extracellular ATP to be an important activator of candidalysin-induced epithelial signalling responses, namely epidermal growth factor receptor (EGFR) and MAPK signalling, which mediate downstream innate immunity during oral epithelial infection. The data provide novel mechanistic insight into the induction of multiple key alarmins and AMPs, important for antifungal defences against *C. albicans*.

## 1. Introduction

*Candida albicans* is a highly prevalent fungal species found living harmoniously within the human body as part of a healthy microbiota [1]. However, under predisposing conditions, such as antibiotic treatment or host immunocompromise, *C. albicans* can infect mucosal tissues and potentially translocate across epithelial barriers to cause life-threatening systemic disease [2,3]. Suitably predisposing conditions may promote *C. albicans* hypha formation [4]; a dynamic, energy-dependent process highly associated with pathogenicity and characterised by the secretion of the *ECE1*-encoded cytolytic peptide toxin, candidalysin [5,6,7,8,9,10,11]. Notably, candidalysin is secreted exclusively by pathogenic *C. albicans* hyphae and not commensal yeast, providing a unique mechanism for host cells to distinguish between potentially harmful and benign species, as well as initiate appropriate immune responses against this microbe.

Oral epithelial cells respond directly to the presence of candidalysin by activating epidermal growth factor receptor (EGFR) [12], a key tyrosine kinase important in cell biology and infection [13]. Consequent MAPK signalling, via the AP-1 transcription factor c-Fos and MAPK phosphatase (MKP)-1 [14,15], as well as the release of inflammatory cytokines IL-1α, IL-1β, IL-36γ, IL-6, GM-CSF and G-CSF [12,14,15,16,17,18,19,20], follows EGFR activation. Candidalysin-induced IL-1α/β and IL-36γ signalling drives expansion of innate TCRαβ^+^ T-cells and consequent IL-17A expression, required for robust antifungal immune responses at the oral mucosa [20,21]. A central theme of this immune response is neutrophil recruitment, which is mediated by candidalysin activity and is a key component for effective defences during oropharyngeal [5], systemic [22] and central nervous system (CNS) *C. albicans* infections [23]. Contrastingly, however, exacerbation of disease and immunopathology has also been observed as a consequence of neutrophil activity during vulvovaginal candidiasis [24,25], suggesting dysregulated immune control at this site.

Though our understanding of the host immune response following *C. albicans* infections has greatly improved in recent years, details of initial activation events at the epithelial surface remain unclear. What is known, however, includes the immediate and early release of alarmins and antimicrobial peptides (AMPs) [26] in response to pathogenic *C. albicans* infection. Alarmins are a group of endogenous molecules secreted in response to cellular stresses that result from sterile inflammation or pathogen mediated injury. As such, alarmins provide warning signals that contribute to immune activation and tissue repair [27]. AMPs on the other hand, largely function to damage cell walls resulting in pathogen lysis, in addition to their immune-modulating abilities [28].

S100 proteins [29,30,31] and defensins [31,32,33,34] are highlighted as two main alarmin and AMP molecules induced by *C. albicans,* functioning to recruit neutrophils [29,30] and permeabilise fungal cell walls [35], respectively. However, information on the specific *C. albicans* factors that trigger the secretion of these molecules, or indeed other alarmins and AMPs, has largely remained elusive. Herein, we identify candidalysin as a potent stimulus of several key alarmins and AMPs including S100A8, human β-defensin (hBD)2, hBD3, the cathelicidin LL37, ATP and reactive oxygen and nitrogen species (ROS/RNS). Additionally, we highlight the importance of extracellular ATP in candidalysin-induced epithelial activation and immune signalling during *C. albicans* infection.

## 2. Materials and Methods

Cell culture: The human buccal epithelial squamous cell carcinoma cell line, TR146 [36], was obtained from the European Collection of Authenticated Cell Cultures (ECACC) and cultured in Dulbecco’s Modified Eagle’s Medium: Nutrient Mixture F12 (DMEM/F12, GIBCO, UK), supplemented with 10% foetal bovine serum and 1% penicillin–streptomycin. At 24 h prior to experimentation, the culture medium was replaced with serum-free medium and maintained until cells were harvested.

*C. albicans* strains: BWP17+CIp30 [37] wild-type (WT), *ece1Δ/Δ (ece1 null)*, *ece1Δ/Δ+ECE1* and *ece1Δ/Δ+ECE1_Δ184-279_* (see Supplementary Table S1 or Reference [5] for full genotype information). *C. albicans* was cultured in YPD medium (1% yeast extract, 2% peptone, 2% dextrose in water) in a non-airtight container, shaken at 200 rpm at 30 °C. Cultures were washed twice in PBS, prior to spectrophotometer analysis and diluted in appropriate culture media to the required concentration. A multiplicity of infection (MOI) of 5 was used for 2 h experiments and MOI 0.005 used with 24 h studies.

Toxins: Candidalysin (SIIGIIMGILGNIPQVIQIIMSIVKAFKGNK) was synthesised by Peptide Protein Research Ltd (UK), solubilised in culture grade water and stored at 10 mg/mL at −20 °C. Nigericin was purchased from Sigma-Aldrich (Cat. No.: 481990-5), reconstituted in ethanol and stored at −20 °C.

Inhibitors: Apyrase (#A6535 Sigma-Aldrich) was solubilised in ddH_2_O to a dilution of 1 mg/mL as recommended by the manufacturer, then used immediately or aliquoted and frozen for storage. Use of Apyrase within two weeks of reconstitution yielded optimum results. Tempol (Tocris #3082) was reconstituted in DMSO, aliquoted and frozen for storage (−20 °C) at 100 mg/mL or diluted into appropriate concentrations for immediate use.

ELISA: Collected supernatants were centrifuged at 13000 rpm for 10 min prior to aliquoting and storage at −80 °C. ELISA kits were purchased from R&D Systems, (S100A8 Duoset kit, Oxfordshire, UK), Elabscience, (LL37, Oxfordshire, UK), LS Bio, (hBD3, Cambridge, UK) or Cusabio, (hBD1, Oxfordshire, UK) and performed according to the manufacturer’s instructions. hBD2 was detected in culture supernatants using the following reagents: goat anti-hBD2 primary antibody (#6500P161G Peprotech), recombinant hBD2 (#300-49, Peprotech), biotinylated goat anti-hBD2 (#500-P161GBt, Peprotech), Streptavidin-HRP, substrate and stop solution (#DY999 R&D Systems). The following was conducted at room temperature with three PBS wash steps between each stage. Briefly, Maxisorp, Nunc 96-well plates were coated with 100 µL of primary antibody and incubated overnight at 4 °C. Wells were blocked with a 1% BSA solution at room temperature for 30 min, prior to addition of 100 µL samples/standards for 1.5 h. 100 µL of secondary antibody was then added at 0.1 µg/mL to wells for 60 min, followed by incubation with streptavidin-HRP (30 min), addition of substrate (5–20 min) and stop solution (50 µL). Analysis was conducted at 450 nm within 30 min.

ATP and ROS detection: Extracellular ATP was detected using an ATP Assay kit (#ab83355 Abcam) according to the manufacturer’s instructions. Briefly, 50 µL standards and samples were added to a 96-well plate suitable for fluorescent analysis (black sides, clear bottom). A reaction mixture containing an ATP converter, probe, buffer and ‘developer mix’ was then added to all wells (50 µL) and incubated away from light at room temperature for 30 min. Analysis was conducted at 535/587 nm within 2 h. 

Extracellular ROS/RNS was detected using the OxySelect In vitro ROS/RNS Assay Kit (#STA-347, Cell Biolabs Inc) and performed as instructed by the manufacturer. Briefly, 50 µL of samples and standards were added in triplicate to a 96-well plate suitable for fluorescent analysis and incubated for 5 min. Addition of 50 µL Catalyst was then followed by a 100 µL Probe solution for 30 min. Fluorescence was analysed at 480 nm excitation / 530 emission. Data are expressed as the amount of relative fluorescent units (RFU) of dichlorodihydrofluorescin DiOxyQ (DCFH-DiOxyQ) or DCF probe detected.

Western blotting: RIPA lysis buffer (50 mM Tris-HCl pH 7.4, 150 mM NaCl, 1 mM EDTA, 1% Triton X-100, 1% sodium deoxycholate, 0.1% SDS) containing protease (Sigma-Aldrich) and phosphatase (Perbio Science) inhibitors was used to lyse cells following experimentation. Cell lysates were incubated in ice for 30 min after harvesting then centrifuged at 13,000 rpm at 4 °C for 10 min to remove debris before transfer to a new tube and storage at −80 °C. Protein concentration was determined using a BCA protein quantitation kit (Perbio Science) and 10 µg of total protein extract was separated on 12% acrylamide SDS-PAGE gels before transferring to nitrocellulose membranes (GE Healthcare). Membranes were incubated with primary (1:1000) and secondary (1:10000) antibodies before addition of immobilon chemiluminescent substrate (Millipore) and exposure to X-ray film (Fiji film). Primary antibodies were all purchased Cell Signalling Technology: pEGFR Y1068 (#3777S), c-Fos (#2250S), pMKP1 (#2857S) or Millipore: α-Actin (#MAB1501).

MTT: MTT was purchased from Sigma-Aldrich (M5655) and prepared to 5 mg/mL in PBS and sterilised through a 0.2 µm filter. TR146 cells were seeded at a density of 2 × 10^5^/mL in a flat-bottom 96-well plate. The next day normal growth media was replaced with serum-free DMEM for 24 h. Serum-free DMEM alone or containing Apyrase or Triton X was added to wells in triplicate for 3 or 25 h. This was followed by the addition of 20 µL MTT to all wells for 3 h at 37 °C. 150 µL of MTT solubilisation solution (50% dimethylformamide, 20 mM HCl, 0.2% glacial acetic acid and 10% SDS) diluted in deionised H_2_O) was then added to all wells and incubated overnight at 37 °C. Absorbance was measured at 620 nm wavelength. Values are calculated as a percentage of control (untreated wells).

Cytokine quantification: A magnetic fluorokine performance MAP cytokine multiplex kit (Bio-techne) was used alongside a Bioplex 200 machine to quantify levels of cytokine from culture supernatants. Antibody beads were purchased from Bio-techne and Bioplex manager 6.1 software was used to determine analyte concentrations.

Statistics: One-way analysis of variance (ANOVA) was used alongside a Bonferroni post hoc test to correct for multiple comparisons. A *p*-value of less than 0.05 was considered significant and represented as *, while *p* < 0.01 = ** and *p* < 0.001 = ***.

## 3. Results

### 3.1. Candidalysin Induces hBD2, hBD3 and LL37 Release during Oral C. Albicans Infection In Vitro

**Figure 1:**
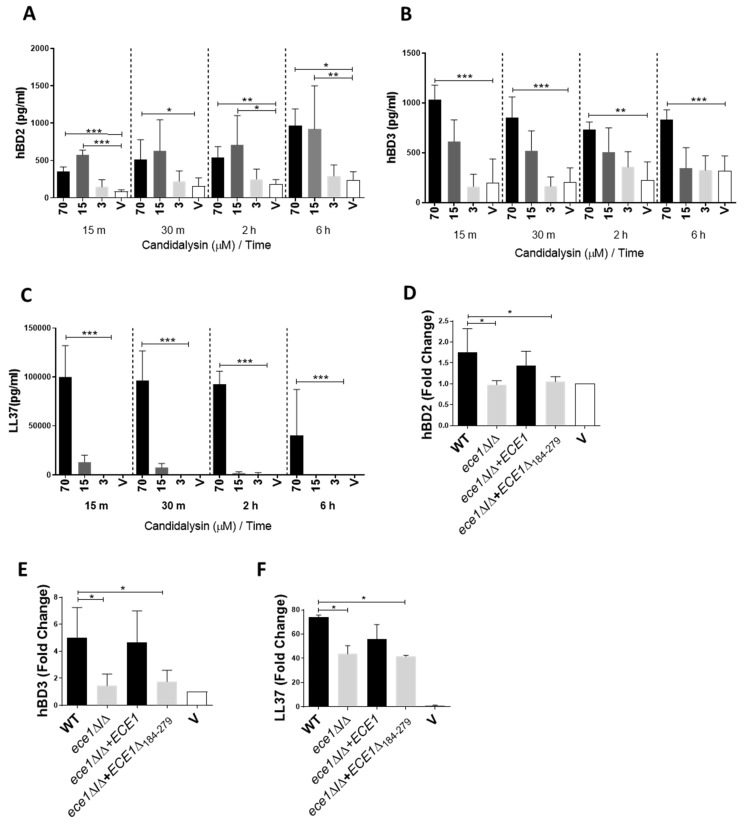
Candidalysin induces secretion of antimicrobial peptides hBD2, hBD3 and LL37. All three molecules were secreted from oral epithelial cells 15 min post candidalysin treatment in a dose-dependent manner (**A**–**C**). These proteins were also secreted following infection with candidalysin-expressing *C. albicans* (**D**–**F** Black bars), but deficiently secreted or not secreted at all in response *C. albicans* mutants lacking candidalysin (**D**–**F** grey bars). Infected cell supernatants were assayed at 24 h post-infection (**D**–**F**) and are expressed as fold change relative to the vehicle control. Data are an average (**A**–**E**) or representative (**F**) of three biological experiments. Unmatched, one-way ANOVA with Bonferroni multiple comparison’s test was used to assess statistical significance. Error bars represent SD, * *p* < 0.05, ** *p* < 0.01, *** *p* < 0.001.

A number of host-derived AMPs are induced during *C. albicans* mucosal infection. To investigate the role of candidalysin in AMP induction, we incubated TR146 oral epithelial cells with synthetic candidalysin or *C. albicans* fungal strains and analysed culture supernatants for hBD2, hBD3 and LL37. All three AMPs were significantly induced by candidalysin in a dose-dependent manner at 15 min post-treatment and remained highly expressed for at least 6 h (Figure 1A–C). Moreover, candidalysin-deficient *C. albicans* strains (ece1Δ/Δ and ece1Δ/Δ+ECE1_Δ184-279_) were incapable of inducing hBD2 and hBD3 (Figure 1D,E (grey bars)) and significantly less inducive of LL37 as compared with wild-type (WT) *C. albicans* (Figure 1F (grey vs black bars)). The *C. albicans* ECE1 recomplemented strain ece1Δ/Δ+ECE1, which produces candidalysin, was capable of near wild-type levels of AMP induction. Notably, hBD1 release was not triggered by candidalysin or *C. albicans* (Supplementary Figure S1A,B).

### 3.2. Candidalysin Promotes ATP, ROS/RNS and S100A8 Release 

**Figure 2:**
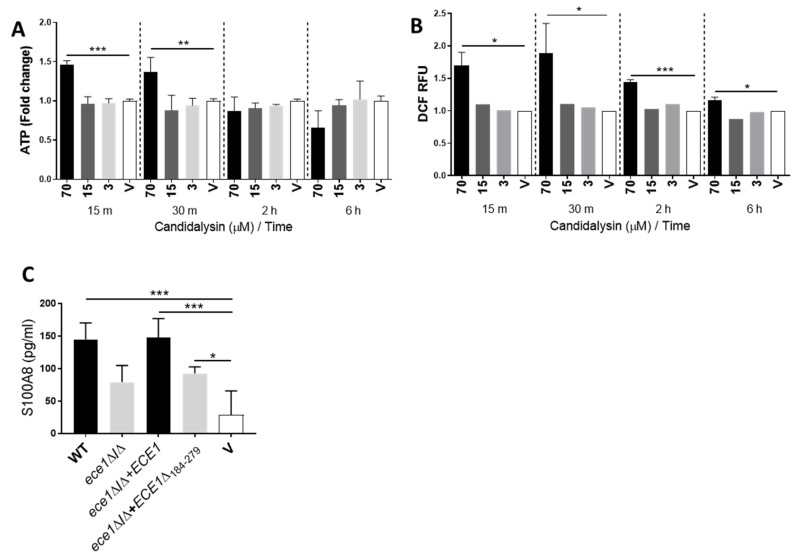
Candidalysin the induces release of ATP, ROS/RNS and S100A8 alarmins. ATP (**A**) and ROS/RNS (**B**) molecules are released from oral epithelial cells following treatment with 70 µM candidalysin. Initial elevated ATP levels are recovered at 2 h post-treatment, whereas increased ROS/RNS levels are maintained for 6 h. At 24 h post-infection, induction of S100A8 by candidalysin-deficient strains is significantly suppressed (**C** (grey bars)) when compared to that of candidalysin-expressing strains (**C** (black bars)). Data are an average of three biological experiments. Unmatched, one-way ANOVA with Bonferroni multiple comparison’s test was used to assess statistical significance. Error bars represent SD, * *p* < 0.05, ** *p* < 0.01, *** *p* < 0.001.

We next assessed the role of candidalysin in inducing the epithelial alarmins ATP, ROS/RNS and S100A8. Given that ATP [38] and ROS [39] are produced by both Candida and epithelial cells, we used candidalysin alone as a stimulus to ensure the detection of host-produced alarmins only. Candidalysin induced the release of ATP (Figure 2A) and ROS/RNS (Figure 2B) in a dose-dependent manner, at 15 and 30 min post-treatment. No secretion of S100A8 was detected upon candidalysin treatment (Supplementary Figure S1C) but, interestingly, induction was observed following *C. albicans* infection (Figure 2C (black bars)), with candidalysin-deficient fungal mutants exhibiting diminished ability to trigger S100A8 release (Figure 2C (grey bars)). A modest and insignificant increase in HMGB1 induction was also observed upon candidalysin treatment at higher doses, but not following *C. albicans* infection (Supplementary Figure S1D,E).

### 3.3. ATP Contributes to Epithelial Cell Activation and Signalling

**Figure 3:**
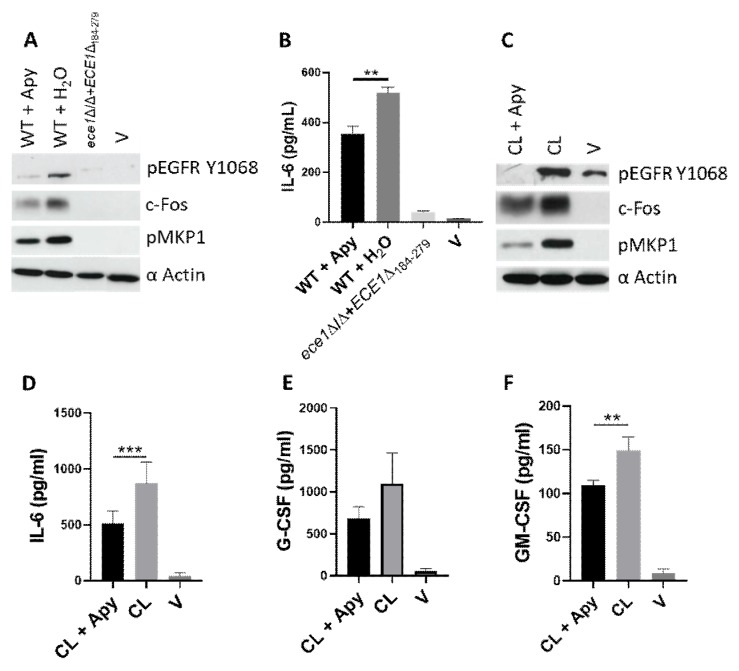
Reduction of available ATP suppresses candidalysin-triggered immune responses. Pre-treatment of cells with the hydrolysing enzyme, Apyrase (10 µM), inhibited *C. albicans*- and candidalysin-induced pEGFR, c-Fos and pMKP1 (**A**,**C**), as well as IL-6 secretion (**B**,**D**). Candidalysin-induced G-CSF (**E**) and GM-CSF (**F**) were also suppressed following Apyrase pre-treatment, though G-CSF inhibition was not statistically significant. Data are representative (**A**–**C**) or an average (**D**–**F**) of three biological experiments. Protein lysates and culture supernatants were harvested at 2 h and 24 h, respectively, post-stimulus. Unmatched, one-way ANOVA with Bonferroni multiple comparison’s test was used to assess statistical significance. Error bars represent SD, ** *p* < 0.01, *** *p* < 0.001.

We previously reported the ability of candidalysin to induce EGFR activation and downstream MAPK signalling [12]. Given that both ATP [40] and ROS [41,42,43] are each capable of triggering EGFR activation, we investigated whether either of these molecules were involved in our model of candidalysin-induced EGFR activation. TR146 oral epithelial cells were incubated with the ATP hydrolysing enzyme, Apyrase, or ROS-scavenger, Tempol, prior to candidalysin treatment or *C. albicans* infection. Reduction in ATP levels via Apyrase treatment significantly suppressed epithelial responses to *C. albicans*, namely pEGFR, c-Fos, pMKP1 (Figure 3A) as well as IL-6 secretion (Figure 3B), but not IL-1α, IL-1β, G-CSF or GM-CSF (Supplementary Figure S2A–D). Similarly, host responses to candidalysin were also significantly suppressed by Apyrase pre-treatment, which impaired pEGFR, c-Fos, pMKP1, IL-6, G-CSF and GM-CSF induction, though inhibition of G-CSF was not statistically significant (Figure 3C–F). However, no effect on candidalysin-induced hBD2, hBD3, LL37 or IL-1α or IL-1β release was observed following Apyrase treatment (Supplementary Figure S2E–I). Notably, ROS inhibition via Tempol had no suppressive effect on *C. albicans*- or candidalysin-induced activation of pEGFR, c-Fos or pMKP1 (Supplementary Figure S3A,B). Given this, reduction in cytokine release was not assessed with Tempol. To ensure that Apyrase activity did not result in an overall loss of cellular fitness, we conducted a mitochondrial metabolic assessment (MTT assay) to determine TR146 cell viability following Apyrase treatment. Cell viability was not affected by Apyrase at 3 h or 25 h post-treatment (Supplementary Figure S3C,D), confirming the importance of extracellular ATP in candidalysin-induced EGFR activation, MAPK signalling and consequent IL-6 induction.

### 3.4. Candidalysin-Induced Responses Are Specific and Not a Consequence of General Pore Formation

**Figure 4:**
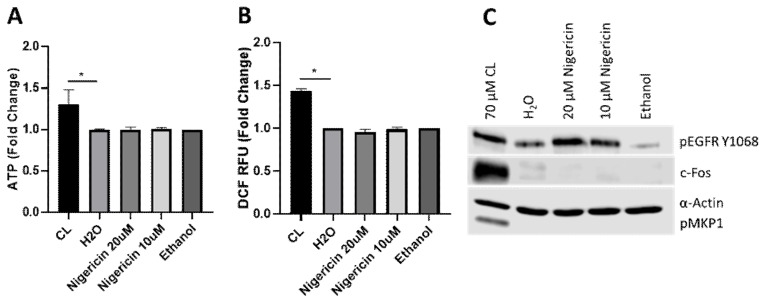
Nigericin and candidalysin do not induce comparable effects. Pre-treatment of oral epithelial cells with nigericin at 20 µM or 10 µM does not induce ATP (**A**), ROS/RNS (**B**), c-Fos or pMKP1 (**C**); epidermal growth factor receptor (EGFR), however, is phosphorylated following nigericin treatment (**C**). Data are an average (**A**, **B**) or representative (**C**) of at least three biological experiments. Culture supernatants and protein lysates were harvested at 15 min and 2 h, respectively, post candidalysin stimulus. Unmatched, one-way ANOVA with Bonferroni multiple comparison’s test was used to assess statistical significance. Error bars represent SD, * *p* < 0.05.

To determine the specificity of candidalysin-induced responses, we compared responses between candidalysin and nigericin, an unrelated pore-forming toxin from the bacterial species *Streptomyces hygroscopicus.* We found that nigericin was unable to induce ATP or ROS/RNS in oral epithelial cells (Figure 4A,B). While EGFR phosphorylation was induced, downstream MAPK components (c-Fos and MKP1) were not activated (Figure 4C). Thus, while nigericin forms pores and may activate EGFR, it does not induce a comparable biological response to that of candidalysin. The data suggest that ATP release (and subsequent downstream responses) is specific to candidalysin and not a consequence of general pore formation. 

## 4. Discussion

This study identifies candidalysin as a potent, hypha-specific inducer of alarmins and AMPs from human oral epithelial cells, and highlights the importance of ATP in initiating candidalysin-induced signalling events required for downstream immune responses.

A number of host protective AMPs and alarmins are induced in response to *C. albicans* infection within a variety of cell types, including human palate [33], vaginal [29] and oesophageal [32] epithelium, as well as human keratinocytes [44], blood-derived neutrophils [45] and the intestinal cell line Caco2 [34]. However, the identity of the specific fungal factors that trigger these alarmins and AMPs is largely unknown. *C. albicans* derived β-glucans [45] and phospholipomannan [44] have been reported as inducers of defensins in neutrophils and keratinocytes, respectively, but as components of the fungal cell wall, they are expressed by both *C. albicans* yeast and hyphae. Thus, whether they are associated with commensal homeostasis or pathogenic immunity is unclear, making it difficult to ascertain their importance during disease and subsequent host-immunity. Herein, we highlight candidalysin as the first hypha-specific *C. albicans* factor identified to trigger the release of multiple alarmins and AMPs in human oral epithelial cells. The data may be useful when considering novel anti-fungal strategies with specific regard to pathogenic *C. albicans* species.

Upon *C. albicans* infection, the epithelial release of hBD2 and hBD3 are wholly attributed, and LL37 and S100A8 partially attributed, to candidalysin activity. Additionally, each molecule, except S100A8, is induced by candidalysin treatment alone. These observations in human oral epithelial cells align with previous studies highlighting the necessity for candidalysin in the induction of murine *BD3* and *S100A* gene transcripts during oropharyngeal [21] and vulvovaginal candidiasis [24]. Candidalysin independent mechanisms of S100A8 and LL37 release may also exist, as we find the absence of candidalysin does not completely abrogate induction of either protein during infection.

Importantly, our findings also reveal an ability for candidalysin to induce epithelial-derived ATP and ROS/RNS alarmins. Contrastingly, the bacterial pore-forming toxin, nigericin, was unable to mimic this function, suggesting that release of alarmins is not a consequence of general pore formation at the epithelial membrane, but a specific and active response to candidalysin. We find initial elevated ATP levels return to normal within 2 h and may reflect the rapid hydrolysis of ATP to ADP/AMP in fuelling biological processes. Indeed, this short-lived elevation appears important in candidalysin-triggered responses, since reduced availability of ATP (via Apyrase) significantly inhibits EGFR activation, MAPK signalling (c-Fos, MKP1) and IL-6 release following infection or toxin treatment. In support of this, ATP-dependent transactivation of EGFR has been reported in airway epithelial cells, which is thought to occur via dual oxidase 1 (DUOX1) and disintegrin and metalloproteinase domain-containing protein (ADAM)-17 [40]. Given that activation of EGFR and downstream signalling are known to support protective anti-*Candida* immunity [5,12,24], the data may demonstrate a key role for ATP in triggering epithelial immune responses against *C. albicans*.

The release of other known candidalysin-induced proteins, such as hBD2, hBD3, LL37, G-CSF, GM-CSF, IL-1α and IL-1β, do not appear to be affected by Apyrase during infection. Thus a combination of factors and underlying redundancy likely exist within the system, with events such as calcium influx, shown to have roles in ROS induction [46] and *Candidalysin*-induced immune responses [12], potentially playing a role.

In addition to direct activation of epithelial cells, other anti-*Candida* functions of alarmins and AMPs are known to exist. These include recruitment of host protective neutrophils (S100 proteins [29,30] and defensins [31]), pathogen membrane permeabilisation (defensins [35] and LL37 [47]), attachment to cell wall proteins to inhibit host binding (LL37 [48]), as well as killing via induction of ionic destabilisation (histatins [49]) and generation of superoxides or hydrogen peroxide [50]. Together, these reports highlight the value of these molecules in anti-*C. albicans* immunity [31].

Herein, we identify a potent, *C. albicans* derived, hypha-specific inducer of alarmins and AMPs, and highlight ATP as an initiator of candidalysin-induced signalling events required for downstream protective immunity. The data allows for the first time, clear delineation of pathogen-specific responses in the context of alarmins and AMPs, providing new considerations to support anti-*Candida* research and investigation.

## Supplementary Materials

The following are available online at https://www.mdpi.com/2073-4409/9/3/699/s1, Figure S1: Candidalysin does not significantly stimulate hBD1, HMGB1 or S100A8 release. Figure S2: Cytokines and AMPs unaffected by Apyrase pre-treatment. Figure S3: ROS is not involved in candidalysin-induced cell signaling. Apyrase does not affect cell viability. Table S1: Full genotypes of the *C. albicans* strains used.
